# Application of the Surface Regression Technique for Enhancing the Input Factors and Responses for Processing Coconut Oil under Vertical Compression

**DOI:** 10.3390/foods13091384

**Published:** 2024-04-30

**Authors:** Abraham Kabutey, Oldřich Dajbych, Aleš Sedláček, Čestmír Mizera, David Herák

**Affiliations:** Department of Mechanical Engineering, Faculty of Engineering, Czech University of Life Sciences Prague, 165 20 Prague, Czech Republic; dajbych@tf.czu.cz (O.D.); aless@tf.czu.cz (A.S.); mizera@tf.czu.cz (Č.M.); herak@tf.czu.cz (D.H.)

**Keywords:** oil extraction, optimal factors, desirability profile, pareto chart, regression models

## Abstract

This study optimized the input processing factors, namely compression force, pressing speed, heating temperature, and heating time, for extracting oil from desiccated coconut medium using a vertical compression process by applying a maximum load of 100 kN. The samples’ pressing height of 100 mm was measured using a vessel chamber of diameter 60 mm with a plunger. The Box–Behnken design was used to generate the factors’ combinations of 27 experimental runs with each input factor set at three levels. The response surface regression technique was used to determine the optimum input factors of the calculated responses: oil yield (%), oil expression efficiency (%), and energy (J). The optimum factors’ levels were the compression force 65 kN, pressing speed 5 mm min^−1^, heating temperature 80 °C, and heating time 52.5 min. The predicted values of the responses were 48.48%, 78.35%, and 749.58 J. These values were validated based on additional experiments producing 48.18 ± 0.45%, 77.86 ± 0.72%, and 731.36 ± 8.04 J. The percentage error values between the experimental and the predicted values ranged from 0.82 ± 0.65 to 2.43 ± 1.07%, confirming the suitability of the established regression models for estimating the responses.

## 1. Introduction

Coconut fruit tree (*Cocos nucifera* L.) belongs to the family *Arecaceae* (Palmae) and the subfamily *Cocoideae*; with its several uses, it is often called the ‘tree of heaven’ or ‘tree of life’ [1,2]. There are mainly two distinct groups of coconut: tall and dwarf. The tall types grow slowly and bear fruits between 6 and 10 years whereas the dwarf varieties bear fruits early around 4 to 5 years [3,4]. The various products of coconut include tender coconut water, copra, coconut oil, raw kernel, coconut cake, coconut toddy, coconut shell and wood-based products, coconut leaves, and coir pith or coconut fiber [3]. Copra, the dried kernel, which is mainly used for oil extraction, contains about 65–75% oil [3,4]. Coconut provides medicinal benefits for millions of people, especially in the tropical and subtropical regions [3,5]. For instance, coconut oil has been used as a therapeutic treatment for Alzheimer’s disease due to its components including lauric acid, ketone bodies, and medium-chain triglycerides [5,6,7]. The oil has been also used for industrial and food-related applications due to its richness in phenolic compounds, which is linked with scavenging activity [2,8,9]. Coconut oil also contains a high concentration of lauric acid (medium-chain fatty acid) which shows good digestibility since the body uses them as an energy source immediately after their consumption [3].

Over the past decade, the global demand and coconut oil production have been increasing due to the potential health benefits and industrial applications [5]. It is estimated that the world consumption of coconut oil is around 3.5 MT/annum, which is accountable for 2.5% of global vegetable oil production [5,10]. Over 70% of the global coconut production comes from the Philippines and Indonesia where the Philippines is the major exporter of coconut oil, accounting for 42% of the world’s exports [10]. 

Extraction methods have a significant effect on the quality of the coconut oil [2]. In tropical and subtropical regions, coconut oil is traditionally produced by crushing and pressing the copra to extract the oil in large mills and the oil is freely available on the market [9,11]. Other oil extraction techniques include fermentation, enzymatic, fresh-dry, and chilling and thawing. With the fresh-dry process, the mechanical screw press is used to extract the oil [2]. The mechanical screw pressing is widely used for oil extraction from various oil-bearing materials due to its several advantages of producing high-quality oil [12,13,14,15,16]. However, its low oil yield and energy consumption are still a concern and researchers are continuously working on improving its operation by examining the input processing factors, namely moisture content, applied pressure, extraction time, and pressing temperature [17,18,19,20,21] as well as the variation of the press components including the nozzle, cylinder mesh, and screw choke ring sizes [13,22,23,24].

Conventionally, most researchers adopt the classical experimental method where one operating factor is changed at a time while the other factors are kept constant [25,26]. Presently, modeling, and optimization techniques such as the Artificial Neural Networks (ANN) [15,18,27,28], Adaptive neuro_fuzzy inference system (ANFIS) [29,30], and Box–Behnken Design (BBD) coupled with Response Surface Methodology (RSM) [28,29,31] have been employed to reduce the several experiments and time consumption and to maximize cost by determining the optimal processing factors in relation to the responses under investigation. Time consumption is key in oil production and has become a huge obstacle to overcome. In data learning techniques, optimization is one of the primary processes. On a daily basis, many researchers in the world provide better solutions to problems by applying complex methods inherent in the family of optimization. However, the optimum solution is still out there to be found in many scientific and engineering problems and food processing applications [32,33]. In the literature, BBD/RSM has been commonly utilized. It is a statistical approach that is efficient and effective for evaluating the experimental outcome [25,34,35,36,37,38]. The RSM is generally applied to the experimental runs designed by the BBD with the factors’ combinations and the corresponding responses to purposely determine the fitting coefficients of the regression polynomial models and to estimate the favorable conditions for validation through additional experiments to confirm the validity of the regression models developed. The significance of the regression models’ coefficients is assessed by high *F*-values or lower *p*-values from the ANOVA regression analysis at either the 0.01% or 0.05% significance level. In addition, the adequacy of each model is determined by evaluating the lack-of-fit of the model (*p*-value > 0.05) and the high value of the coefficient of determination (R^2^).

It is based on this background that the present study aimed to determine the optimal processing conditions (compression force, pressing speed, heating temperature, and heating time) under a vertical compression process (as described in Section 2.6) for estimating the mass of oil, oil yield, and oil extraction efficiency with the corresponding energy demand of desiccated coconut medium and to validate the optimal processing conditions based on the response surface regression technique based on BBD.

## 2. Materials and Methods

### 2.1. Sample and Experimental Conditions

In total, 25 kg of desiccated coconut medium was obtained from the Farmet Company, Česká Skalice, Czech Republic. The material was kept in a laboratory temperature of 22.4 ± 0.72 °C and humidity of 23.33 ± 0.58%.

### 2.2. Determination of Sample Moisture Content

The sample moisture content was determined using the conventional oven method of temperature of 105 °C and drying time of 17 h [39]. The electronic balance (KERN and SOHN 440–35, Balingen, Germany) with an accuracy of 0.01 g was used for the measurement of the sample weights. The sample moisture content was calculated to be 2.5 ± 0.1% w.b. according to Equation (1) [40].

(1) $$MC=\left(\frac{m_{bf}-m_{af}}{m_{bf}}\right)\cdot 100$$

where MC is the moisture content in wet basis (%), $m_{bf}$ is the mass of the sample before drying, and $m_{af}$ is the mass of the sample after oven drying.

### 2.3. Determination of the Oil Content of the Sample

The sample oil content was determined using the Soxhlet extraction procedure [41,42]. According to the procedure, approximately 11 g of the sample was ground in a mini grinder. The ground sample was put into a thimble and cotton wool was placed atop. The thimble was inserted into the Soxhlet extractor, which was then connected to a 500 mL round bottom flask containing 250 mL of petroleum ether. The setup was placed under a heating source at 60 °C and the solvent was heated to reflux for 24 h. The extracted oil was left in the oven at 50 °C for 4 h to remove the residual solvent. The electronic balance (KERN and SOHN AEJ 200–4CM, Balingen, Germany) with an accuracy of 0.0001 g was used for the sample weight measurement. The experiment was repeated twice and the results were averaged. The sample oil content of 61.88 ± 0.42 (%) was calculated according to Equation (1) by modifying the variables where the numerator $m_{bf}$ represented the mass of flask and extracted oil, $m_{af}$ represented the mass of empty flask, and the denominator $m_{bf}$ represented the mass of the sample.

### 2.4. Box–Behnken Experimental Design

**Figure 1:**
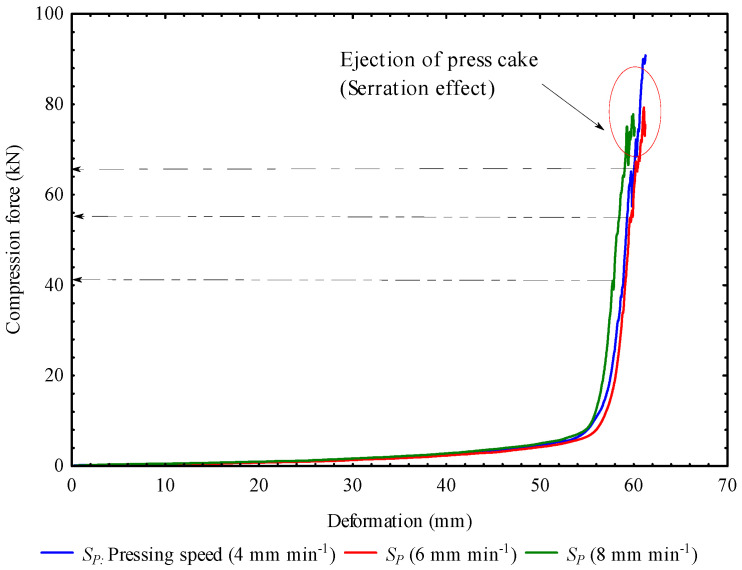
Control experiments of the force–deformation curves of the desiccated coconut medium showing the maximum force and the serration effect in relation to pressing speeds.

A Box–Behnken Design (BBD) was used to generate 27 experimental runs of the factors’ combination (Table 1). The levels of compression force were 45, 55, and 65 kN. The pressing speeds were 4, 6, and 8 mm/min. The heating temperatures were 50, 65, and 80 °C and the heating times were 30, 45, and 60 min. The levels of the compression force and speeds were set based on control experiments (Figure 1) to determine the maximum compression force and speed without the serration effect (circled in red), which affects oil output and energy efficiency.

Conventionally, four input factors with three levels will produce 81 experimental runs and, when repeated thrice, will give 243 experiments, which is time consuming and energy intensive; hence the essence of employing the BBD to minimize the several experiments and still achieve the optimum results. The BBD can be described mathematically according to Equation (2) [34,35,43]:

(2) $$Y=\beta_{0}+\sum_{i=1}^{k}\beta_{i}X_{i}+\sum_{i=1}^{k}\beta_{ii}X_{i}^{2}+\sum_{i_{1_{<j}}}^{k}\sum_{j}^{k}\beta_{ij}X_{i}X_{j}$$

where Y is the response variables; $\beta_{0},\beta_{i}$, $\beta_{ii}$, and $\beta_{ij}$ are the regression coefficients of the intercept, linear, quadratic, and interaction terms, respectively; $X_{I}$ and $X_{j}$ are the independent variables and k is the number of factors. The factors’ levels were coded from −1 (low value) to +1 (high value) with 0 being the center value as given in Equation (3) [35,43], as follows:

(3) $$x_{i}=\frac{X_{i}-X_{0}}{∆X}$$

where $x_{i}$ is the coded value of the i^th^ variable, $X_{i}$ is the uncoded value of the i^th^ test variable, $X_{0}$ is the uncoded value of the i^th^ test variable at the center point, and ∆X is the step-change in the real value of the variable i corresponding to the variation in a unit for the dimensionless value of the variable i.

### 2.5. Samples Pretreatment

The oven equipment (MEMMERT GmbH + Co. KG, Buechenbach, Germany) was used for the pretreatment of the samples at heating temperatures between 50 and 80 and heating times between 30 and 60 min. These ranges were set to examine their effects on the responses.

### 2.6. Compression Tests

**Figure 2:**
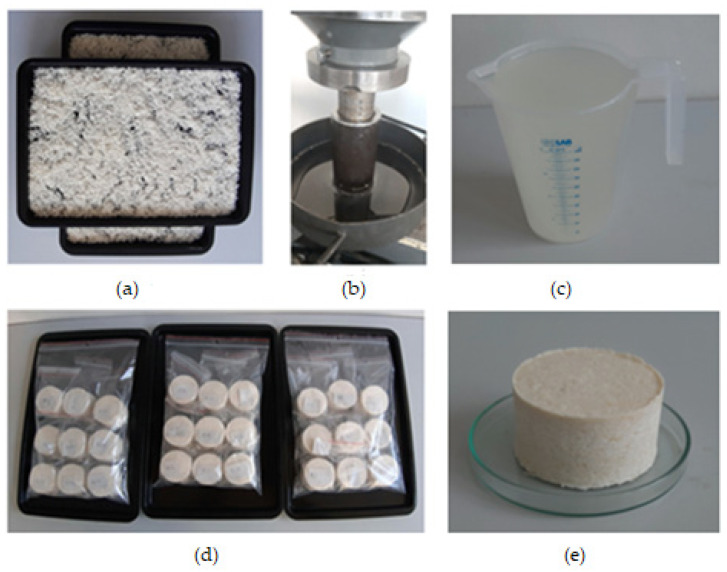
(**a**) Coconut desiccated medium spread out in a tray before heating, (**b**) compression test of a measured sample with the output oil, (**c**) extracted crude coconut oil in a beaker, (**d**) compressed samples of the 27 experimental runs, and (**e**) a compressed sample in petri dish.

**Figure 3:**
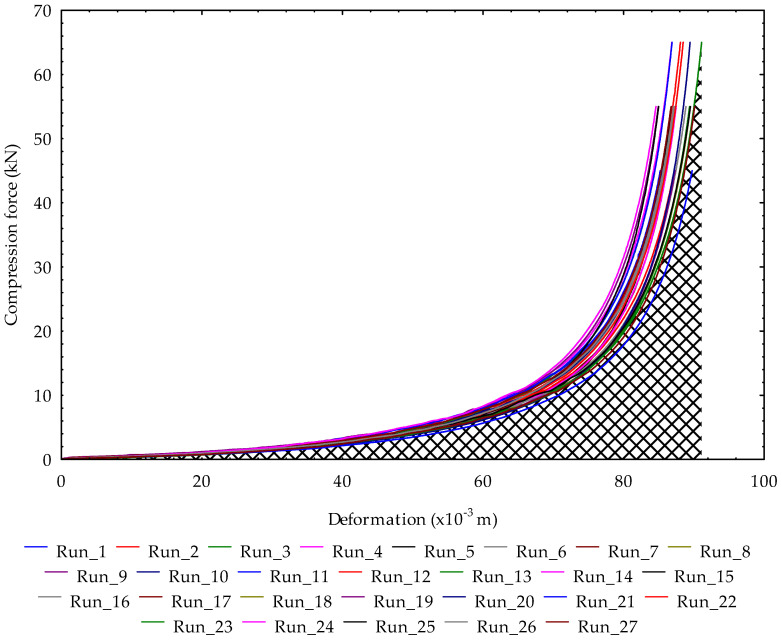
Force–deformation curves of the 27 experimental runs (area under the curve represents the deformation energy for obtaining the oil).

The compression tests were performed using the universal compression testing machine (TEMPOS spol. s.r.o., Opava, Czech Republic (Machine Service), a ZDM 50, VEB Werkstoffprüfmaschinen, Leipzig, Germany) of a maximum load of 500 kN and a pressing vessel of diameter 60 mm with a plunger (Figure 2). The initial pressing height of the sample was measured at 100 mm representing an initial weight of 135.82 g as a constant. Each compression test followed the BBD factors’ combination (Table 1). The force–deformation curves data (Figure 3) were used to calculate the responses, namely the mass of oil, oil yield, oil expression efficiency, energy, and hardness.

### 2.7. Oil Yield

The oil yield was calculated as the ratio of the mass of oil to the mass of the sample multiplied by 100 using Equation (4) [34,44]:

(4) $$O_{Y}=\left[\left(\frac{M_{O}}{M_{S}}\right)\cdot 100\right]$$

where $O_{Y}$ is the oil yield (%), $M_{O}$ is the mass of oil (g) calculated as the difference between the weights of initial sample $M_{S}$ (g) and press cake (g).

### 2.8. Oil Expression Efficiency

The oil expression efficiency was calculated as the ratio of oil yield to that of the percentage oil content using Equation (5) [42,45]:

(5) $$O_{EE}=\left[\left(\frac{O_{Y}}{O_{C}}\right)\cdot 100\right]$$

where $O_{EE}$ is the oil expression efficiency (%) and $O_{C}$ is the sample oil content (%) determined by Soxhlet extraction method as described in Section 2.3.

### 2.9. Deformation Energy

The deformation energy $E_{N}$ was calculated based on the trapezoidal rule using Equation (6) [46,47,48,49], as follows:

(6) $$E_{N}=\sum_{n=0}^{n=i-1}\left[\left(\frac{{F_{R}}_{n+1}+{F_{R}}_{n}}{2}\right)\cdot \left({D_{X}}_{n+1}-{D_{X}}_{n}\right)\right]$$

where $E_{N}$ is the energy (J), ${F_{R}}_{n+1}+{F_{R}}_{n}$ and ${D_{X}}_{n+1}-{D_{X}}_{n}$ are the forces (N) the deformation (mm), *n* is the number of data points, and *i* is the number of sections in which the axis deformation was divided.

### 2.10. Samples Hardness

The hardness of the coconut cakes samples was calculated using Equation (7) [47]:

(7) $$H_{D}=\frac{F_{R}}{D_{X}}$$

where $H_{D}$ is the hardness (N/mm), $F_{R}$ is the compression force (N), and $D_{X}$ is the deformation (mm).

### 2.11. Statistical Analysis of Experimental Data

The experimental data were analyzed by employing the response surface regression technique at a 5% significance level using STATISTICA 13 [50].

## 3. Results and Discussion

### 3.1. Observed Responses

The calculated responses namely the mass of oil, oil yield, oil expression efficiency, energy, and hardness including the obtained deformation values are presented in Table 1. The experimental runs 1–9, 10–18, and 19–27 represented blocks 1, 2, and 3, respectively. For block 1, the factors’ combination (force: 65 kN, pressing speed: 4 mm/min, heating temperature: 45 °C and heating time: 45 min) representing run 2 produced the highest mass of oil output of 64.83 g. For block 2, the mass of oil output of 64.563 g was the highest for the factors’ combination (force: 65 kN, pressing speed: 6 mm/min, heating temperature: 65 °C and heating time: 30 min) representing run 11. The factors’ combination (force: 65 kN, pressing speed: 6 mm/min, heating temperature: 80 °C and heating time: 45 min) obtained the highest mass of oil output of 64.92 g for block 3, representing run 22. The corresponding values of percentage oil yield, oil expression efficiency, deformation energy, deformation, and hardness for experimental runs 2, 11, and 22 ranged from 47.53% to 47.80%, 77.24% to 76.82%, 723.49 to 746.83 J, 86.89 to 88.48 mm, and 0.73 to 0.75 kN/mm. It was observed that the factors’ combination of compression force: 65 kN, pressing speeds: 4 and 6 mm/min, heating temperatures: 45 and 80 °C, and heating times: 30 and 45 min produced the optimal factors for processing oil from desiccated coconut medium under linear pressing. This observation is the essence of the application of the response surface regression technique to statistically determine the optimum factors combination, which is further discussed in Section 3.2.

The hardness of the coconut-pressed cakes provides information on their compressive strength for briquette production for energy purposes. The values ranged from 0.5 to 0.75 kN mm^−1^. The experimental runs 2, 4, 11, 13, 20, and 22 showed high compressive strength or stability of the densified briquettes from desiccated coconut medium in relation to the factors’ combination. The obtained force–deformation curves from the 27 experimental runs are illustrated in Figure 3. The area under the force–deformation curve is the deformation energy based on the trapezoidal rule (Equation (6)).

### 3.2. ANOVA Analysis and Established Regression Models for O_Y_, O_EE_, and E_N_

**Figure 4:**
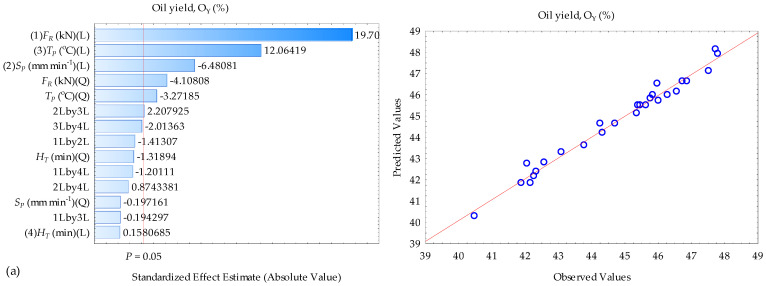

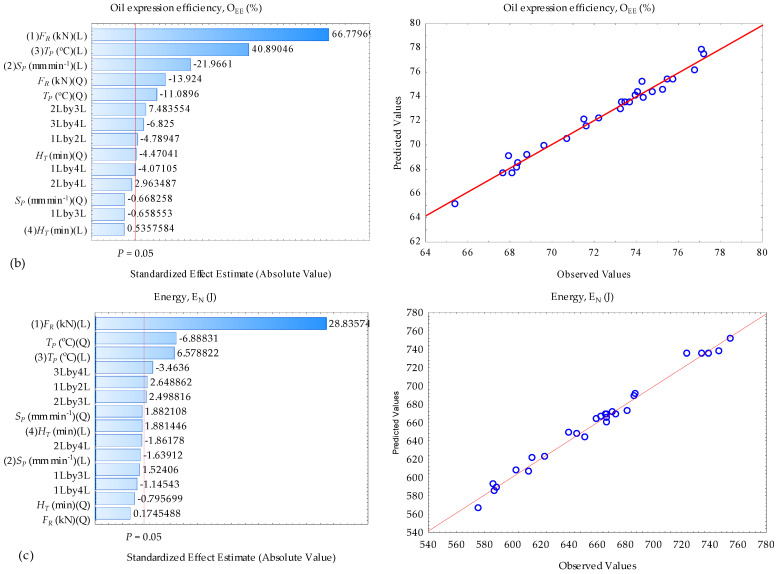
Pareto chart of standardized effects and observed versus predicted values for (**a**) O_Y_, (**b**) O_EE_, and (**c**) E_N_, showing the significance of the factors and their interactions; L: Linear and Q: Quadratic.

The ANOVA results of the responses oil yield, oil expression efficiency, and energy are given in Table 2, Table 3 and Table 4. For oil yield and oil expression efficiency (Table 2 and Table 3), all the coefficients of the effect variables/input factors and their interactions including the intercept were significant (*p* < 0.05) except that of the linear, quadratic, and interaction terms of the pressing speed, heating temperature, force and heating temperature, speed and heating temperature, and speed and heating time showed non-significant (*p* > 0.05). In the case of energy (Table 4), the linear and quadratic terms of the pressing speed and heating temperature as well as the force and heating temperature, force and heating time, and speed and heating time indicated non-significant outcomes (*p* > 0.05) whereas the rest of the terms including the intercept produced significant (*p* < 0.05) outcomes. The significant coefficient terms were included in the regression models described in equations 8, 9, and 10. The models’ adequacy, however, was assessed based on the lack of fit *p*-values, which were non-significant (*p* > 0.05) with high coefficients of determination (R^2^) values ranging between 0.981 and 0.988, indicating that the determined models were suitable for predicting the observed responses. The pareto charts and the plots of the observed versus the predicted (Figure 4) further explain the significance and adequacy (the red line *p* = 0.05 or data points closer to the red line) of the regression models established in Equations (8)–(10).

(8) $$O_{Y}=45.49+2.37\cdot F_{R}-0.74\cdot F_{R}^{2}-0.78\cdot S_{P}+1.45\cdot T_{P}-0.59\cdot T_{P}^{2}-0.24\cdot H_{T}^{2}-0.29\cdot F_{R}\cdot S_{P}+0.46\cdot F_{R}\cdot H_{T}-0.42\cdot T_{P}\cdot H_{T}$$

(9) $$O_{EE}=73.51+3.83\cdot F_{R}-1.20\cdot F_{R}^{2}-1.26\cdot S_{P}+2.35\cdot T_{P}-0.95\cdot T_{P}^{2}-0.38\cdot H_{T}^{2}-0.48\cdot F_{R}\cdot S_{P}+0.74\cdot F_{R}\cdot H_{T}-0.68\cdot T_{P}\cdot H_{T}$$

(10) $$E_{N}=668.68+68.61\cdot F_{R}+15.65\cdot T_{P}-24.58\cdot T_{P}^{2}+10.92\cdot F_{R}\cdot S_{P}\;+10.30\cdot S_{P}{\cdot T}_{P}-14.27\cdot T_{P}\cdot H_{T}$$

### 3.3. Determined Optimal Factors’ Combination for the Responses

**Figure 5:**
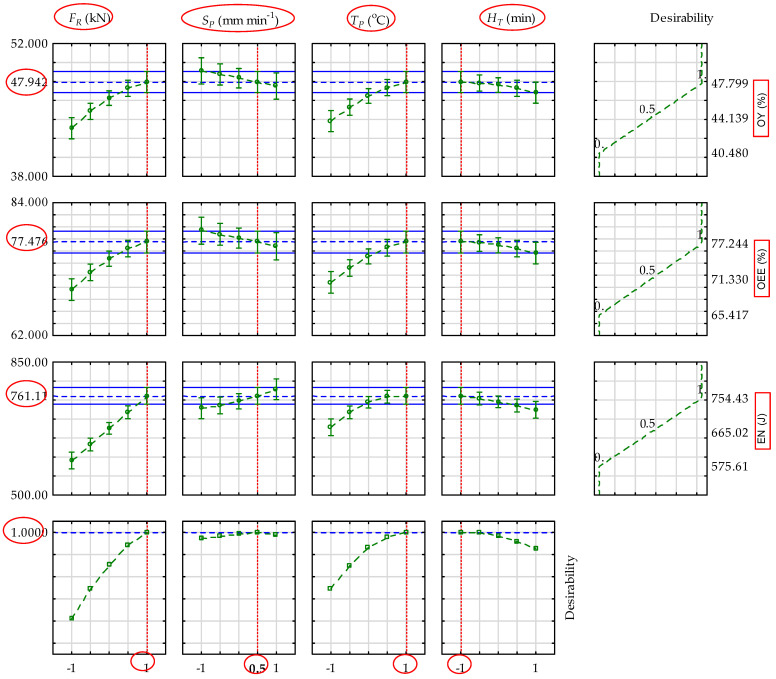
Predicted values for oil yield, O_Y_ (%), oil expression efficiency, $O_{EE}$ (%), and energy, E_N_ (J) with the dashed red line indicating the optimum factors’ levels: $F_{R}$: force; $S_{P}$: speed; $T_{P}$: temperature; and $H_{T}$: heating time) with a strong desirability value of 1.

The optimal values (blue dotted line) of the responses oil yield, oil expression efficiency, and energy and their corresponding optimal input factor levels as well as their desirability values (blue dotted line) are displayed in Figure 5. The optimal factors combinations (red dotted line) for extracting oil from desiccated coconut medium under vertical compression process were found to be $F_{R}$: force (+1 (65 kN)); $S_{P}$: speed (+0.5 (7 mm min^−1^)); $T_{P}$: temperature (+1 (80 °C)); and $H_{T}$: heating time (−1 (30 min)) with a higher desirability of 1. However, the display of each response showed the optimal factors combination to be $F_{R}$: force (+1 (65 kN)); $S_{P}$: speed (−0.5 (5 mm min^−1^)); $T_{P}$: heating temperature (+1 (80 °C)) and $H_{T}$: heating time (+0.5 (52.5 min)) for $O_{Y}$: oil yield and $O_{EE}$: oil expression efficiency. For $E_{N}$: energy, the optimal factor combinations were $F_{R}$: force (+1 (65 kN)); $S_{P}$: speed (+1 (8 mm min^−1^)); $T_{P}$: heating temperature (+1 (80 °C)); and $H_{T}$: heating time (0 (45 min)).

### 3.4. Validation of the Regression Models

The determined optimal levels were validated by conducting additional experiments and the results are presented in Table 5. The predicted values of oil yield, oil expression efficiency, and energy were 48.48%, 78.35%, and 749.58 J based on the regression models (equations 8 to 10). Their validated values were 48.18 ± 0.45%, 77.86 ± 0.72%, and 731.36 ± 8.04 J. The percentage error values between the predicted and the experimental ranged from 0.82 ± 0.65 to 2.43 ± 1.07%, indicating that the determined regression models are adequate for estimating the observed responses.

### 3.5. Established Findings in Relation to Other Studies

The input factors examined were the compression force, pressing speed, heating temperature, and heating time. The compression force is related to pressure or stress. The optimal force of 65 kN represents a pressure of approximately 23 MPa, which was calculated as the ratio of the force to that of the area of the pressing vessel [49]. According to Karaj and Muller [13], for optimization of oil recovery, pressure is the most interesting variable to monitor since higher pressure leads to higher temperature generation resulting into higher oil recovery efficiency. In addition, when temperature increases or heating time is prolonged, the tendency is for the oil to flow more readily from the oil-bearing cells [19]. Rotational/pressing speed also has an effect on the production of oil yield. Higher speed would reduce the residence time and thus result in less chance for oil to flow from the seeds [44,51]. In other words, at higher speeds, the viscosity of the oil thus remains lower, resulting in less pressure build-up and more oil content in press cake [13,16,52]. Moreover, the BBD approach is a type of response surface technique used to maximize the response of a process by determining the correlation between the input factors and the measured responses [25,28,29,31,34,38]. For instance, Kandar and Akil [25] studied three independent process variables including the molding temperature, time, and pressure. The optimum values for the compression parameters were 200 °C, 3 min, and 30 bar to yield 48.902 kJ/m^2^ impact strength for flax-reinforced biocomposites. Chanioti and Tzia [34] also found an oil yield of 11.03% from olive pomace with ultrasound-assisted extraction at optimal conditions of a temperature of 60 °C, solid/liquid ratio of 1/12 g/mL, and particle size of 0.5 mm. Kok et al. [38] applied the BBD with RSM to determine the optimal factors of pressure, temperature, and virgin coconut oil (VGO) mass ratio percentage as co-extract for obtaining xanthones of mangosteen (*Garcinia mangostana*) pericarp with supercritical carbon dioxide extraction. The authors found the highest extraction yield of (31%, 28.2 mg xanthone/g extract) at optimal extraction conditions of 70 °C, 430 bar, and 40% VGO. The study approach and the findings established provide valuable information for processing coconut oil under the vertical compression process.

## 4. Conclusions

A percentage oil yield of 48.18 ± 0.45% and oil expression efficiency of 77.86 ± 0.72% were achieved for processing coconut desiccated medium under the determined optimum input factors combinations of compression force of 65 kN, pressing speed of 5 mm/min, heating temperature of 80 °C, and heating time of 52.5 min. The corresponding energy requirement was 731.36 ± 8.04 J. Regression models for the responses were established at a significant level of 5% which were adequate for estimation based on the models’ lack-of-fit *p*-value, which proved to be non-significant (*p*-value > 0.05). The higher compressive strength/hardness of the coconut press cakes was obtained at a compression force of 65 kN, pressing speed of 6 or 8 mm/min, heating temperature of 65 °C, and heating time of 30 or 45 min. This indicates that coconut press cakes obtained after oil extraction could be blended with other agricultural residues to produce briquettes for biomass energy utilization. The study findings provide valuable information for the development of complex models to describe the oil extraction processes. Future studies should describe the mathematical and relaxation models of coconut desiccated medium under the uniaxial loading process by varying the pressing vessel diameters and initial sample pressing heights at the optimum compression force and pressing speed. A non-linear compression process involving a mechanical screw press should be considered to determine the oil expression efficiency, residual oil content in the press cake, and specific energy consumption for processing coconut desiccated medium.

## Figures and Tables

**Table 1:** Box–Behnken Design of four factor combination with 27 experimental runs and their observed responses.

Run	$F_{R}$ kN	$S_{P}$, mm min^−1^	$T_{P}$, °C	$H_{T}$, min	$M_{O}$, g	$O_{Y}$, %	$O_{EE}$, %	$E_{N}$, J	$D_{X}$, mm	$H_{D}$ kN, mm^−1^
1	45 (−1)	4 (−1)	65 (0)	45 (0)	57.84	42.59	68.82	622.76	85.23	0.53
2	65 (1)	4 (−1)	65 (0)	45 (0)	64.83	47.73	77.14	746.83	88.09	0.74
3	45 (−1)	8 (1)	65 (0)	45 (0)	56.87	41.87	67.67	586.69	84.44	0.53
4	65 (1)	8 (1)	65 (0)	45 (0)	62.26	45.84	74.08	754.43	86.86	0.75
5	55 (0)	6 (0)	50 (−1)	30 (−1)	57.13	42.06	67.98	611.58	86.77	0.63
6	55 (0)	6 (0)	80 (1)	30 (−1)	62.45	45.98	74.31	662.98	87.22	0.63
7	55 (0)	6 (0)	50 (−1)	60 (1)	59.45	43.77	70.74	651.39	87.45	0.63
8	55 (0)	6 (0)	80 (1)	60 (1)	62.49	46.01	74.35	645.71	89.41	0.62
9	55 (0)	6 (0)	65 (0)	45 (0)	61.96	45.62	73.72	673.13	84.94	0.65
10	45 (−1)	6 (0)	65 (0)	30 (−1)	57.25	42.15	68.12	588.56	85.53	0.53
11	65 (1)	6 (0)	65 (0)	30 (−1)	64.56	47.53	76.82	739.65	86.89	0.75
12	45 (−1)	6 (0)	65 (0)	60 (1)	57.5	42.34	68.42	602.31	87.18	0.52
13	65 (1)	6 (0)	65 (0)	60 (1)	63.45	46.72	75.49	734.52	91.1	0.71
14	55 (0)	4 (−1)	50 (−1)	45 (0)	60.71	44.70	72.23	640.00	87.47	0.63
15	55 (0)	8 (1)	50 (−1)	45 (0)	57.42	42.28	68.32	613.96	84.99	0.65
16	55 (0)	4 (−1)	80 (1)	45 (0)	63.67	46.88	75.76	666.67	88.86	0.62
17	55 (0)	8 (1)	80 (1)	45 (0)	62.88	46.30	74.82	681.82	86.76	0.63
18	55 (0)	6 (0)	65 (0)	45 (0)	61.63	45.38	73.33	666.10	89.5	0.61
19	45 (−1)	6 (0)	50 (−1)	45 (0)	54.98	40.48	65.42	575.61	87.33	0.52
20	65 (1)	6 (0)	50 (−1)	45 (0)	61.59	45.35	73.28	687.13	89.46	0.73
21	45 (−1)	6 (0)	80 (1)	45 (0)	58.53	43.09	69.64	586.85	89.75	0.50
22	65 (1)	6 (0)	80 (1)	45 (0)	64.92	47.80	77.24	723.49	88.48	0.73
23	55 (0)	4 (−1)	65 (0)	30 (−1)	63.26	46.58	75.27	659.65	89.39	0.62
24	55 (0)	8 (1)	65 (0)	30 (−1)	60.22	44.34	71.65	671.28	84.6	0.65
25	55 (0)	4 (−1)	65 (0)	60 (1)	62.17	45.77	73.97	686.28	89.5	0.61
26	55 (0)	8 (1)	65 (0)	60 (1)	60.12	44.26	71.53	667.21	87.1	0.63
27	55 (0)	6 (0)	65 (0)	45 (0)	61.75	45.46	73.47	666.81	90.1	0.61

$F_{R}$: compression force; $S_{P}$: pressing speed; $T_{P}$: heating temperature; $H_{T}$: heating time; $O_{Y}$: oil yield; $O_{EE}$: oil expression efficiency, $E_{N}$: energy; $D_{X}$: deformation; and $H_{D.}$: hardness. The coded values from +1 to −1 represent the high, center, and low values.

**Table 2:** ANOVA regression model parameters for oil yield, $O_{Y}$ (%).

Effect	Model $O_{Y}$ (%) ^a^	Std. Err. Pure Err.	Sum of Squares	df	Mean Square	*F*-Value	*p*-Value
Intercept	45.49	0.07	106.54	14	7.61	43.79	0.00 *
$F_{R}$	2.37	0.04	67.45	1	67.45	4459.53	0.00 *
$F_{R}^{2}$	–0.74	0.05	2.93	1	2.93	193.88	0.01 *
$S_{P}$	–0.78	0.04	7.30	1	7.30	482.51	0.00 *
$S_{P}^{2}$	–0.04	0.05	0.01	1	0.01	0.45	0.57 **
$T_{P}$	1.45	0.04	25.29	1	25.29	1672.03	0.00 *
$T_{P}^{2}$	–0.59	0.05	1.86	1	1.86	122.98	0.01 *
$H_{T}$	0.02	0.04	0.00	1	0.00	0.29	0.65 **
$H_{T}^{2}$	–0.24	0.05	0.30	1	0.30	19.98	0.05 *
$F_{R}$* $S_{P}$	–0.29	0.06	0.35	1	0.35	22.94	0.04 *
$F_{R}$* $T_{P}$	–0.04	0.06	0.01	1	0.01	0.43	0.58 **
$S_{P}$* $T_{P}$	–0.25	0.06	0.25	1	0.25	16.57	0.06 **
$F_{R}$* $H_{T}$	0.46	0.06	0.85	1	0.85	56.00	0.02 *
$S_{P}$* $H_{T}$	0.18	0.06	0.13	1	0.13	8.78	0.10 **
$T_{P}$* $H_{T}$	–0.42	0.06	0.70	1	0.70	46.58	0.02 *
Residual			2.08	12	0.17		
Lack of fit			2.05	10	0.21	13.59	0.07 **
Pure Error			0.03	2	0.04		
Total			108.63	26			

$F_{R}$: compressive force; $S_{P}$: pressing speed; $T_{P}$: heating temperature; $H_{T}$: heating time; ^a^: coefficient of determination (R^2^) = 0.981; df: degrees of freedom; Std. Err.: standard error; *: Significant (*p* < 0.05); **: Non-significant (*p* > 0.05).

**Table 3:** ANOVA regression model parameters for oil expression efficiency, $O_{EE}$ (%).

Effect	Model $O_{EE}$ (%) ^b^	Std. Err. Pure Err.	Sum of Squares	df	Mean Square	*F*-Value	*p*-Value
Intercept	73.51	0.11	278.24	14	19.87	43.79	0.00 *
$F_{R}$	3.83	0.06	176.14	1	176.14	4459.53	0.00 *
$F_{R}^{2}$	−1.20	0.09	7.66	1	7.66	193.88	0.01 *
$S_{P}$	−1.26	0.06	19.06	1	19.06	482.51	0.00 *
$S_{P}^{2}$	−0.06	0.09	0.02	1	0.02	0.45	0.57 **
$T_{P}$	2.35	0.06	66.04	1	66.04	1672.03	0.00 *
$T_{P}^{2}$	−0.95	0.09	4.86	1	4.86	122.98	0.01 *
$H_{T}$	0.03	0.06	0.01	1	0.01	0.29	0.65 **
$H_{T}^{2}$	−0.38	0.09	0.79	1	0.79	19.98	0.05 *
$F_{R}$* $S_{P}$	−0.48	0.10	0.91	1	0.91	22.94	0.04 *
$F_{R}$* $T_{P}$	−0.07	0.10	0.02	1	0.02	0.43	0.58 **
$S_{P}$* $T_{P}$	−0.40	0.10	0.65	1	0.65	16.57	0.06 **
$F_{R}$* $H_{T}$	0.74	0.10	2.21	1	2.21	56.00	0.02 *
$S_{P}$* $H_{T}$	0.29	0.10	0.35	1	0.35	8.78	0.10 **
$T_{P}$* $H_{T}$	−0.68	0.10	1.84	1	1.84	46.58	0.02 *
Residual			5.45	12	0.45		
Lack of fit			5.37	10	0.54	13.59	0.07 **
Pure Error			0.08	2	0.04		
Total			283.68	26			

$F_{R}$: compressive force; $S_{P}$: pressing speed; $T_{P}$: heating temperature; $H_{T}$: heating time; ^b^: coefficient of determination (R^2^) = 0.981; df: degrees of freedom; Std. Err.: standard error; *: Significant (*p* < 0.05); **: Non-significant (*p* > 0.05).

**Table 4:** ANOVA regression model parameters for energy, $E_{N}$ (J).

Effect	Model $E_{N}$ (%) ^c^	Std. Err. Pure Err.	Sum of Squares	df	Mean Square	*F*-Value	*p*-Value
Intercept	668.68	2.23	66,768.48	14	4769.18	70.21	0.00 *
$F_{R}$	68.61	1.12	56,482.09	1	56,482.09	3774.583	0.00 *
$F_{R}^{2}$	0.62	1.68	2.07	1	2.07	0.138	0.01 **
$S_{P}$	−3.90	1.12	182.50	1	182.50	12.196	0.00 **
$S_{P}^{2}$	6.72	1.68	240.62	1	240.62	16.080	0.57 **
$T_{P}$	15.65	1.12	2939.98	1	2939.98	196.473	0.00 *
$T_{P}^{2}$	−24.58	1.68	3223.10	1	3223.10	215.393	0.01 *
$H_{T}$	4.48	1.12	240.45	1	240.45	16.069	0.65 **
$H_{T}^{2}$	−2.84	1.68	43.01	1	43.01	2.874	0.05 **
$F_{R}$* $S_{P}$	10.92	1.93	476.61	1	476.61	31.851	0.04 *
$F_{R}$* $T_{P}$	6.28	1.93	157.78	1	157.78	10.544	0.58 **
$F_{R}$* $H_{T}$	−4.72	1.93	89.12	1	89.12	5.956	0.06 **
$S_{P}$* $T_{P}$	10.30	1.93	424.15	1	424.15	28.345	0.02 *
$S_{P}$* $H_{T}$	−7.67	1.93	235.45	1	235.45	15.735	0.10 **
$T_{P}$* $H_{T}$	−14.27	1.93	814.90	1	814.90	54.458	0.02 *
Residual			815.14	12	67.93		
Lack of fit			785.21	10	78.52	5.25	0.17 **
Pure Error			29.93	2	14.96		
Total			67,583.62	26			

$F_{R}$: compressive force; $S_{P}$: pressing speed; $T_{P}$: heating temperature; $H_{T}$: heating time; ^c^: coefficient of determination (R^2^) = 0.988; df: degrees of freedom; Std. Err.: Standard Error; *: Significant (*p* < 0.05); **: Non-significant (*p* > 0.05).

**Table 5:** Validation of optimized operating factors for the observed responses; O_Y_, O_EE_, and E_N_.

Unit of Observed Responses	* Predicted Values (Equations (8)–(10))	Experimental Values (Validated)	Percentage Error (%)
$O_{Y}\;$(%)	48.48	48.18 ± 0.45	0.82 ± 0.65
$O_{EE}\;$(%)	78.35	77.86 ± 0.72	0.82 ± 0.65
$E_{N}\;$(J)	749.58	731.36 ± 8.04	2.43 ± 1.07

* $F_{R}$: compressive force (+1 (65 kN)); $S_{P}$: pressing speed (−0.5 (5 mm min^−1^)); $T_{P}$: heating temperature (+1 (80 °C)), $H_{T}$: heating time (+0.5 (52.5 min)) for $O_{Y}$: oil yield and $O_{EE}$: oil expression efficiency; for $E_{N}$: energy − $F_{R}$: compressive force (+1 (65 kN)); $S_{P}$: pressing speed (+1 (8 mm min^−1^)); $T_{P}$: heating temperature (+1 (80 °C)), $H_{T}$: heating time (0 (45 min)).

## Data Availability

The original contributions presented in the study are included in the article, further inquiries can be directed to the corresponding author.
